# Distinct Drivers of Core and Accessory Components of Soil Microbial Community Functional Diversity under Environmental Changes

**DOI:** 10.1128/mSystems.00374-19

**Published:** 2019-10-01

**Authors:** Ximei Zhang, Eric R. Johnston, Yaosheng Wang, Qiang Yu, Dashuan Tian, Zhiping Wang, Yanqing Zhang, Daozhi Gong, Chun Luo, Wei Liu, Junjie Yang, Xingguo Han

**Affiliations:** a Key Laboratory of Dryland Agriculture, MOA, Institute of Environment and Sustainable Development in Agriculture, Chinese Academy of Agricultural Sciences, Beijing, China; b Biosciences Division, Oak Ridge National Laboratory, Oak Ridge, Tennessee, USA; c National Hulunber Grassland Ecosystem Observation and Research Station, Institute of Agricultural Resources and Regional Planning, Chinese Academy of Agricultural Sciences, Beijing, China; d Key Laboratory of Ecosystem Network Observation and Modeling, Institute of Geographic Sciences and Natural Resources Research, Chinese Academy of Sciences, Beijing, China; e State Key Laboratory of Vegetation and Environmental Change, Institute of Botany, Chinese Academy of Sciences, Beijing, China; f Shanghai Majorbio Bio-pharm Biotechnology Co., Ltd., Shanghai, China; g Key Laboratory of Plant Resources and Beijing Botanical Garden, Institute of Botany, Chinese Academy of Sciences, Beijing, China; University of California San Diego

**Keywords:** accessory community gene, core community gene, grassland, increased precipitation, microbial diversity, nitrogen deposition

## Abstract

It is a central ecological goal to explore the effects of global change factors on soil microbial communities. The vast functional gene repertoire of soil microbial communities is composed of both core and accessory genes, which may be governed by distinct drivers. This intuitive hypothesis, however, remains largely unexplored. We conducted a 5-year nitrogen and water addition experiment in the Eurasian steppe and quantified microbial gene diversity via shotgun metagenomics. Nitrogen addition led to an 11-fold increase in the abundance (based on quantitative PCR [qPCR]) of ammonia-oxidizing bacteria, which have mainly core community genes and few accessory community genes. Thus, nitrogen addition substantially increased the relative abundance of many core genes at the whole-community level. Water addition stimulated both plant diversity and microbial respiration; however, increased carbon/energy resources from plants did not counteract increased respiration, so soil carbon/energy resources became more limited. Thus, water addition selected for microorganisms with genes responsible for degrading recalcitrant soil organic matter. Accordingly, many other microorganisms without these genes (but likely with other accessory community genes due to relatively stable average microbial genome size) were selected against, leading to the decrease in the diversity of accessory community genes. In summary, nitrogen addition primarily affected core community genes through nitrogen-cycling processes, and water addition primarily regulated accessory community genes through carbon-cycling processes. Although both gene components may significantly respond as the intensity of nitrogen/water addition increases, our results demonstrated how these common global change factors distinctly impact each component.

**IMPORTANCE** Our results demonstrated increased ecosystem nitrogen and water content as the primary drivers of the core and accessory components of soil microbial community functional diversity, respectively. Our findings suggested that more attention should be paid to certain components of community functional diversity under specific global change conditions. Our findings also indicated that microbial communities have adapted to nitrogen addition by strengthening the function of ammonia oxidization to deplete the excess nitrogen, thus maintaining ecosystem homeostasis. Because community gene richness is primarily determined by the presence/absence of accessory community genes, our findings further implied that strategies such as maintaining the amount of soil organic matter could be adopted to effectively improve the functional gene diversity of soil microbial communities subject to global change factors.

## Abstract (precis)

Our results demonstrated increased ecosystem nitrogen and water content as the primary drivers of the core and accessory components of soil microbial community functional diversity, respectively. Our findings suggested that more attention should be paid to certain components of community functional diversity under specific global change conditions. Our findings also indicated that microbial communities have adapted to nitrogen addition by strengthening the function of ammonia oxidization to deplete the excess nitrogen, thus maintaining ecosystem homeostasis. Because community gene richness is primarily determined by the presence/absence of accessory community genes, our findings further implied that strategies such as maintaining the amount of soil organic matter could be adopted to effectively improve the functional gene diversity of soil microbial communities subject to global change factors.

## INTRODUCTION

The increasing extent and intensity of human activities are resulting in many different types of environmental change, such as climate warming, altered precipitation patterns, and nitrogen (N) deposition (1–4). The biodiversity and functioning of a particular natural ecosystem are often colimited by multiple environmental factors, such as the supplies of energy and nutrients. Anthropogenic environmental changes can alter these environmental restrictions and lead to changes in both biodiversity and ecosystem functioning (5–7). Thus, it is a central goal in current ecological research to explore the influences of environmental changes on belowground microbial communities (8–10). However, the majority of previous studies have focused on microbial taxonomic diversity rather than their functional gene diversity, because molecular and computational methods allowing for the assessment of functional gene diversity have only recently become widely accessible (9, 11, 12).

The genes within the genomes of microbial communities have differing ecological importance. For example, within the bacterial communities of human guts, some genes have functions necessary for a bacterium to thrive in the gut context, and they are present in nearly all gut bacterial species, and thus, they are most important and belong to the “minimal gut genome” (13). In particular, there are two types of genes in the “minimal gut genome”: those required in all bacteria (housekeeping), such as the genes with functions that are part of main metabolic pathways (e.g., amino acid synthesis, central carbon metabolism), and those potentially specific for the gut, such as the genes with functions involved in adhesion to the host proteins (e.g., collagen, fibrinogen). In addition, many genes carried by certain community members are involved in the homeostasis of the whole ecosystem (13), such as those responsible for the biodegradation of complex sugars and glycans harvested from the host diet and/or intestinal lining (e.g., pectin and sorbitol, sugars that are omnipresent in fruits and vegetables but that are not absorbed or poorly absorbed by humans), and these genes are present in the gut metagenomes of nearly all individuals. These genes, together with the genes of the “minimal gut genome”, make up the “minimal gut metagenome” (13). Thus, the genes of the “minimal gut metagenome” are traditionally thought to be the core community genes, and accordingly, the other genes present in only some gut metagenomes can be taken as the accessory community genes. The “minimal gut metagenome” is operationally defined as the common components that are shared by different gut metagenomes (13); thus, as the number of gut metagenome samples increases, the number of core community genes will decrease and that of accessory community genes will increase.

Similar to human gut microbial communities, functional genes within the microbial communities of soil systems can conceivably be classified into either the core or accessory components (13). The core component of a soil microbial community includes not only the genes essential for an individual microorganism (such as the genes responsible for DNA replication and RNA transcription, which are present in almost all species) but also those necessary for the homeostasis of the whole soil system (which are carried by many species). In contrast, accessory community genes are necessary for living under specific conditions, such as these responsible for antibiotic resistance. Thus, genes that are common among soil samples belonging to a distinct field study should primarily be core community genes, and those observed in only some samples should primarily be accessory community genes. Accordingly, the functional gene diversity/composition of soil microbial communities is codetermined by the relative abundance of core community genes and the presence or absence of accessory community genes (12). In particular, gene richness is primarily determined by the presence or absence of accessory community genes, while abundance-based compositional variation may be largely determined by changes in the relative abundance of core community genes. Both the core and accessory community genes may respond significantly to a given environmental change factor, especially when the environmental change intensity is sufficiently large. Nevertheless, due to their distinctive attributes, the relative abundances of core community genes and the presence or absence of accessory community genes may be primarily determined by distinct environmental factors, and thus, either component may respond uniquely to different global change factors. However, to date, this intuitive hypothesis has been poorly explored.

Microorganisms are the key drivers of both soil carbon (C)- and N-cycling processes (5, 14). In many terrestrial ecosystems, ammonia oxidization is the rate-limiting step among various N-cycling processes, including N fixation, mineralization, nitrification, and denitrification (15). Furthermore, ammonia-oxidizing bacteria (AOB) are often found to be the most sensitive N-cycling functional group under many different global change factors (16–18). Since AOB acquire energy through oxidizing ammonia, a sharp increase in soil NH_4_^+^-N content caused by N addition/deposition will lead to a substantial increase in AOB abundance. AOB have very small genomes, with mainly core community genes and very few accessory community genes; therefore, substantial increases in AOB abundance will lead to dramatic shifts in the measured relative abundances of core community genes of entire belowground microbial communities (15). The majority of soil microorganisms in most terrestrial ecosystems acquire C (energy) resources through catabolism of different types of soil organic matter (SOM), which have various degrees of decomposability and require different mechanisms for decomposition (19, 20). If a specific global change factor (e.g., overgrazing) decreases the provision of soil labile C resources, microorganisms with genes that enable the degradation of relatively recalcitrant SOM could be selected for. Accordingly, microorganisms that rely primarily on labile substrates without such recalcitrant SOM degradation genes could be selected against. However, microorganisms may have relatively stable genome sizes, and species without these recalcitrant SOM degradation genes may carry many other accessory genes (21). Thus, a decrease in labile C resources will lead to a decline in the richness of accessory genes in the entire microbial community. In contrast, if certain global change factors (e.g., revegetation) were to increase the content of soil labile C resources, the richness of accessory community genes will recover; in other words, more available energy can support a wider repertoire of accessory community genes.

The semiarid temperate steppe of northern China is an important part of the Eurasian grassland biome (22). Water (W) and N represent two of the most common environmental limitations in this ecosystem; both biodiversity and ecosystem functioning are particularly sensitive to N enrichment and increased precipitation in this area (23–26). Thus, we conducted a 5-year field experiment with N addition, W addition, and their combination (N and W addition) in this ecosystem. We employed whole-community shotgun metagenome sequencing analyses to quantify soil microbial functional gene diversity and composition. We aimed to test the following hypotheses. (i) N addition will primarily affect the relative abundance of core genes of soil microbial communities by stimulating soil ammonia oxidization processes. (ii) W addition will primarily affect the presence or absence of accessory genes of microbial communities through stimulating plant productivity and increasing soil labile C resources.

## RESULTS

### N and W addition affected soil, plant, and microbial indices.

N and W addition significantly affected different soil, plant, and microbial indices (Table 1). In particular, the N-addition treatments (both N and NW [concurrent N and W addition]) significantly increased SOM content by 21.3% relative to the non-N-addition treatments (both control and W) (*P* < 0.05). They also significantly increased soil available N content by 36.6%, while decreasing soil pH by 0.67, on average (Table 1). W-addition treatments (both W and NW) significantly increased soil water content from 7% to 11% and increased plant species richness by 25.3% (Table 1). In addition, the N-addition treatments significantly increased ammonia oxidization potential by 559% and decreased microbial respiration by 6.0% (Table 1). W-addition treatments increased microbial respiration by 10.8%. W addition decreased fungal relative abundance from 1.34% to 0.78% throughout the microbial community and increased bacterial relative abundance from 93.62% to 94.54%; however, it had no significant effect on the relative abundance of *Archaea* (Table 1).

**TABLE 1:** Effects of experimental treatments on the biotic and abiotic indices

Biotic or abiotic index	Value [mean (SE)] for index for the following treatment:				*P* value of split-plot ANOVA^*a*^				
	Control	W	N	NW	Block	N	Block*N	W	N*W
SOM content (g kg^−1^ soil)	10.30 (0.94)	10.00 (1.18)	12.62 (1.14)	12.00 (0.59)	0.159	**0.048**	0.528	0.595	0.850
Soil total N content (g kg^−1^ soil)	2.72 (0.15)	2.70 (0.14)	2.50 (0.07)	2.50 (0.01)	0.252	**0.020**	0.60	0.801	0.801
Soil available N content (mg kg^−1^ soil)	13.47 (0.92)	19.68 (1.61)	26.39 (2.62)	25.88 (6.03)	0.396	**0.045**	0.355	0.381	0.308
Soil water content (kg kg^−1^ soil)	0.07 (0.01)	0.11 (0.01)	0.07 (0.01)	0.11 (0.01)	0.362	0.734	0.264	**<0.001**	0.592
Soil pH	7.22 (0.07)	7.32 (0.10)	6.43 (0.03)	6.78 (0.17)	0.488	**0.004**	0.742	0.118	0.360
Aboveground plant biomass (g m^−2^)	45.93 (13.02)	46.38 (1.93)	47.67 (5.18)	77.58 (15.36)	0.938	0.395	0.058	0.103	0.112
Plant species richness	11.25 (0.48)	14.25 (1.44)	9.50 (0.65)	11.75 (1.65)	0.457	0.231	0.084	**0.013**	0.633
Bacterial 16S rRNA gene abundance (10^10^/g soil)	1.11 (0.15)	1.10 (0.14)	0.91 (0.11)	1.22 (0.15)	0.543	0.813	0.459	0.332	0.302
Bacterial relative abundance (%)	93.59 (0.52)	95.03 (0.34)	93.65 (0.18)	94.04 (0.27)	0.061	0.127	0.231	**0.009**	0.087
Archaeal relative abundance (%)	5.23 (0.60)	4.26 (0.19)	4.85 (0.09)	5.10 (0.21)	0.144	0.453	0.354	0.249	0.065
Fungal relative abundance (%)	1.18 (0.16)	0.71 (0.17)	1.50 (0.2)	0.86 (0.17)	0.932	0.267	0.659	**0.021**	0.674
Microbial respiration (mg CO_2_ kg^−1^ day^−1^)	40.56 (1.07)	43.39 (0.97)	36.71 (1.06)	42.20 (1.16)	0.246	**0.031**	0.187	**0.002**	0.214
Ammonia oxidization potential (NO_2_^−^-N mg g^−1^ soil h^−1^)	1.52 (0.60)	1.27 (0.44)	9.48 (0.90)	8.91 (1.08)	0.021	**<0.001**	0.274	0.473	0.779

a *P* values of <0.05 are shown in boldface type. Block*N represents the interactive effect of block and N, and N*W represents the interactive effect of N and W.

### N addition affected the relative abundance of core community genes, while W addition governed the presence/absence of accessory community genes.

After rarifying to exclude the influence of unequal metagenomic sequencing, 4,647 core community genes were identified in each of the 16 soil samples, and they collectively accounted for 99.83% ± 0.02% (mean ± standard error) of relative abundance of all functional gene annotations in each sample. A total of 1,317 ± 77 accessory community genes were identified in each sample, and they collectively took up 0.17% ± 0.02% of relative abundance of all functional gene annotations in each sample.

**FIG 1:**
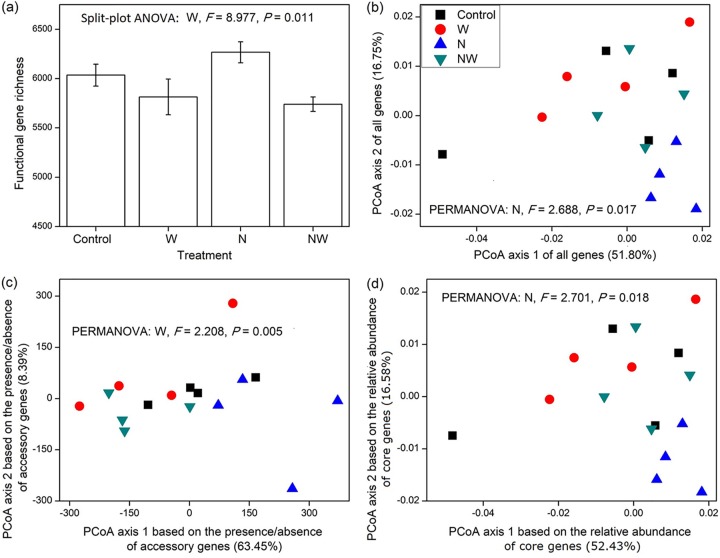
Effects of experimental treatments on soil microbial community functional gene richness (a) and their functional composition based on either all genes, accessory community genes, or core community genes (b to d). Error bars represent the standard errors (four replicates). The values in parentheses on the axes of panels b to d show the percentage of community compositional variation explained by the principal-component analysis (PCoA) axes. For clarity, only the significant (*P* < 0.05) statistical results are shown in the figure.

W-addition treatments significantly decreased functional gene richness by 6.1% in comparison to the non-W-addition treatments (*P* < 0.05; Fig. 1a). In particular, the gene richness of 18 out of 25 COG (Clusters of Orthologous Groups) categories decreased with W addition (*P* < 0.10; see Table S1 in the supplemental material). Gene richness is primarily determined by the presence or absence of accessory community genes rather than the relative abundance of core community genes; therefore, this result indicates that W addition primarily affected accessory genes rather than core community genes. This conclusion was further confirmed by the result that W addition had a significant effect (*P* < 0.01; Fig. 1c) on the compositional variation based on only the presence or absence of accessory community genes (Bray-Curtis distance based on the presence or absence of accessory community genes; those not observed in all sample metagenomes).

10.1128/mSystems.00374-19.4 TABLE S1 Effects of experimental treatments on the gene richness of each COG category. Download Table S1, XLSX file, 0.01 MB. Copyright © 2019 Zhang et al. 2019 Zhang et al. This content is distributed under the terms of the Creative Commons Attribution 4.0 International license.

N addition significantly altered the composition of soil microbial community functional genes (*P* < 0.05; Fig. 1b). In particular, N addition increased the relative abundances of COG categories that are responsible for carbohydrate transport and metabolism, coenzyme transport and metabolism, and transcription and accordingly decreased the relative abundances of COG categories responsible for “replication, recombination, and repair,” cell motility, “secondary metabolites biosynthesis, transport, and catabolism,” signal transduction mechanisms, and defense mechanisms (*P* < 0.05; Table S2). Since core community genes collectively make up 99.83% ± 0.02% of total relative abundance, functional gene composition (Bray-Curtis distance based on all genes) should be determined primarily by the relative abundance of core community genes rather than by the presence or absence of accessory community genes; therefore, this result indicates that N addition primarily affected the core community genes rather than the accessory community genes. This conclusion was further confirmed by the result that N addition had a significant effect (*P* < 0.05; Fig. 1d) on the compositional variation based on the relative abundance of core community genes only (Bray-Curtis distance based on the relative abundance of genes observed in all samples).

10.1128/mSystems.00374-19.5 TABLE S2 Effects of experimental treatments on the relative abundance of each COG category. Download Table S2, XLSX file, 0.01 MB. Copyright © 2019 Zhang et al. 2019 Zhang et al. This content is distributed under the terms of the Creative Commons Attribution 4.0 International license.

In contrast to the significant effects on functional gene richness and composition, these treatments had no significant effect on the operational taxonomic unit (OTU) richness of soil bacterial communities (using 16S sequences derived from metagenomes). This result obtained from shotgun metagenome sequencing was further confirmed with pyrosequencing of bacterial 16S rRNA genes (see Fig. S1 in the supplemental material). However, both N and W addition significantly affected the taxonomic composition (phylum level) of the soil microbial community (*P* < 0.05; Fig. S1). In particular, N addition significantly increased the relative abundances of the phyla *Gemmatimonadetes*, *Nitrospirae*, and *Ascomycota* and decreased the relative abundances of *Acidobacteria* and *Planctomycetes*. W addition significantly increased the relative abundances of the phyla *Chloroflexi*, *Planctomycetes*, *Proteobacteria*, and *Zygomycota* and decreased the relative abundances of *Firmicutes* and *Ascomycota* (Table S3). In addition, the sample-to-sample distances (β-diversity) derived from functional gene composition correlated significantly with those obtained using taxonomic composition (Mantel test, *r *= 0.585, *P* < 0.001).

10.1128/mSystems.00374-19.1 FIG S1 Effects of experimental treatments on the microbial OTU richness and composition (a and b) quantified with shotgun metagenome sequencing and the bacterial OTU richness (c) quantified with 454 pyrosequencing. Error bars represent one standard error (four replicates). The values in the parentheses on the axes in panel b represent the percentage of community compositional variation explained by the PCoA axes. For clarity, only the significant (*P* < 0.05) statistical results are shown in the figure. Download FIG S1, DOCX file, 0.2 MB. Copyright © 2019 Zhang et al. 2019 Zhang et al. This content is distributed under the terms of the Creative Commons Attribution 4.0 International license.

10.1128/mSystems.00374-19.6 TABLE S3 Effects of experimental treatments on the relative abundances of different taxonomic groups. Download Table S3, XLSX file, 0.01 MB. Copyright © 2019 Zhang et al. 2019 Zhang et al. This content is distributed under the terms of the Creative Commons Attribution 4.0 International license.

### W addition promoted the deterministic process of ecological filtering and affected the relative abundances of organic C degradation genes.

**FIG 2:**
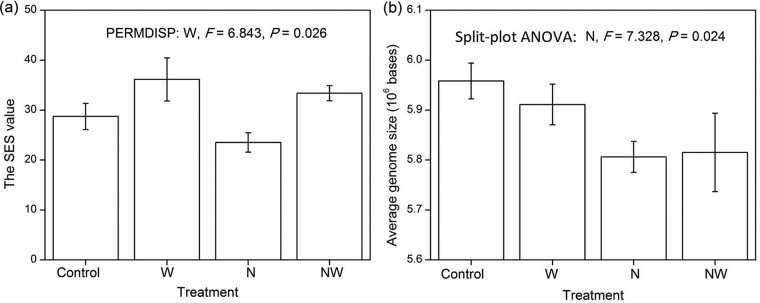
Effects of experimental treatments on the SES value (a) and the average genome size (b) of soil microbial communities. Error bars represent one standard error (four replicates). For clarity, only the significant (*P* < 0.05) statistical results are shown in the figure.

When the functional gene data of each treatment were individually analyzed, at least a marginally significant difference was found between the observed community similarity and the expected community similarity in a completely random assemblage (from the null model) under all four treatments (*P* < 0.10; Table S4). In particular, the observed similarity exceeded that of the expected similarity, indicating the deterministic process of ecological filtering (rather than stochastic processes) as a more important driver of functional gene diversity. Furthermore, when the functional gene data of all four treatments were analyzed together, the W-addition treatments caused significantly larger SES (standard effect size) values than the non-W-addition treatments (*P* < 0.05; Fig. 2a), indicating that W addition promoted the deterministic process of ecological filtering.

10.1128/mSystems.00374-19.7 TABLE S4 Significant test of the difference between observed community similarity and expected community similarity. Download Table S4, XLSX file, 0.01 MB. Copyright © 2019 Zhang et al. 2019 Zhang et al. This content is distributed under the terms of the Creative Commons Attribution 4.0 International license.

Consistent with the stimulation effect of W addition on the ecological filtering process, W addition decreased the relative abundances of many genes responsible for the degradation of labile, simple carbohydrates and increased the relative abundances of most genes involved in the degradation of the relatively recalcitrant SOM (amino acids and aromatic compounds; Table 2). In contrast, N addition did not exert a consistent effect on the genes for the degradation of SOM with differing decomposability (Table S5).

**TABLE 2:** Effects of water (W) addition on the relative abundances of SOM degradation genes

Gene KO and function annotation^*a*^	Relative abundance^*b*^ of SOM degradation gene for the following treatment:				*P*^*c*^	Effect^*d*^
	Control	W	N	NW		
Degradation of labile carbohydrates						
K01178: EC 3.2.1.3; glucoamylase	44.62 (1.73)	40.62 (1.27)	47.68 (0.94)	41.58 (0.98)	<0.001	↓
K01813: *rhaA*; l-rhamnose isomerase	9.17 (0.41)	9.72 (0.45)	8.47 (0.31)	9.52 (0.17)	0.003	↑
K01193: EC 3.2.1.26, *sacA*;beta-fructofuranosidase	7.53 (0.92)	6.83 (0.26)	10.07 (0.52)	7.62 (0.49)	0.004	↓
K01195: *uidA*, GUSB; beta-glucuronidase	37.64 (2.56)	33.69 (0.58)	39.34 (1.16)	33.97 (0.38)	0.005	↓
K01818: *fucI*; l-fucose isomerase	6.78 (0.28)	5.74 (0.3)	8.23 (0.63)	7.29 (0.56)	0.006	↓
K02783: PTS-Gut-EIIC, *srlA*; PTS system, glucitol/sorbitol-specific IIC component	3.14 (0.32)	2.77 (0.27)	3.55 (0.22)	2.82 (0.38)	0.024	↓
K00882: *fruK*; 1-phosphofructokinase	28.4 (2.94)	25.57 (1.54)	32.3 (1.42)	29.06 (1.66)	0.025	↓
K01628: *fucA*; l-fuculose-phosphate aldolase	20.42 (1.36)	19.77 (0.35)	22.56 (0.68)	18.71 (1.65)	0.031	↓
K00011: EC 1.1.1.21, AKR1; aldehyde reductase	0.97 (0.33)	0 (0)	0.94 (0.32)	0.35 (0.35)	0.036	↓
K13057: *treT*; trehalose synthase	42.79 (3.16)	40.63 (1.03)	45.11 (2.07)	39.01 (1.14)	0.037	↓
K07026: EC 3.1.3.70; mannosyl-3-phospho-glycerate phosphatase	0.40 (0.40)	0 (0)	0.71 (0.44)	0 (0)	0.056	↓
K01840: *manB*; phosphomannomutase	116.59 (2.83)	113.32 (3.19)	117.21 (1.5)	113.18 (0.89)	0.064	↓
K00696: EC 2.4.1.14; sucrose-phosphate synthase	3.56 (0.43)	3.06 (0.31)	3.59 (0.14)	2.53 (0.39)	0.068	↓
K00966: GMPP; mannose-1-phosphate guanylyltransferase	44.08 (1.22)	41.62 (1.31)	48.09 (1.81)	43.53 (2.68)	0.085	↓

Degradation of amino acids						
K00253: IVD, *ivd*; isovaleryl-CoA dehydrogenase	28.75 (0.92)	33.18 (1.11)	26.06 (1.6)	29.92 (1.64)	0.005	↑
K00822: EC 2.6.1.18; beta-alanine-pyruvate transaminase	21.33 (1.40)	25.49 (0.94)	20.65 (0.22)	22.69 (1.24)	0.013	↑
K07514: EHHADH; enoyl-CoA hydratase/ 3-hydroxyacyl-CoA dehydrogenase/ 3,2-*trans*-enoyl-CoA isomerase	0.26 (0.26)	1.07 (0.04)	0 (0)	0.28 (0.28)	0.023	↑
K01848: EC 5.4.99.2A, *mcmA1*; methylmalonyl -CoA mutase, N-terminal domain	300.22 (3.28)	280.3 (6.82)	283.65 (4.11)	280.58 (5.74)	0.029	↓
K01782: *fadJ*; 3-hydroxyacyl-CoA dehydrogenase/ enoyl-CoA hydratase/ 3-hydroxybutyryl-CoA epimerase	53.94 (1.46)	60.75 (1.04)	60.28 (1.48)	60.34 (1.4)	0.053	↑
K00020: EC 1.1.1.31, *mmsB*;3-hydroxyisobutyrate dehydrogenase	49.40 (1.57)	54.52 (1.41)	53.48 (1.63)	53.68 (1.12)	0.063	↑
K01969: EC 6.4.1.4B; 3-methylcrotonyl-CoA carboxylase beta subunit	92.26 (3.67)	97.43 (3.03)	86.54 (1.36)	92.22 (3.1)	0.080	↑
K00263: EC 1.4.1.9; leucine dehydrogenase	49.94 (1.56)	48.05 (1.44)	52.15 (1.05)	49.87 (0.24)	0.089	↓

Degradation of aromatic compounds						
K03379: EC 1.14.13.22; cyclohexanone monooxygenase	30.02 (0.81)	34.33 (1.03)	31.18 (1.07)	31.99 (0.74)	0.021	↑
K01053: EC 3.1.1.17, *gnl*, RGN; gluconolactonase	76.96 (1.91)	84.07 (0.84)	73.18 (2.34)	77.58 (2.96)	0.035	↑
K01856: *catB*; muconate cycloisomerase	6.02 (0.39)	5.29 (0.26)	5.71 (0.29)	5.05 (0.3)	0.047	↓
K05711: *hcaB*; 3-dihydroxy-2,3- dihydrophenylpropionate dehydrogenase	2.30 (0.20)	1.94 (0.03)	2.99 (0.41)	2.26 (0.37)	0.055	↓
K00151: *hpaE*, *hpcC*; 5-carboxymethyl-2-hydroxymuconic semialdehyde dehydrogenase	19.51 (0.73)	20.86 (0.86)	22.34 (0.98)	23.25 (0.24)	0.081	↑
K10219: *ligC*; 2-hydroxy-4-carboxy- muconate semialdehyde hemiacetal dehydrogenase	4.95 (0.17)	6.02 (0.28)	4.91 (0.43)	4.93 (0.25)	0.095	↑

a Category, KEGG accession no., enzyme nomenclature designation, and enzyme or gene and enzyme and function annotation are shown. Abbreviations: PTS, phosphotransferase; EIIC, enzyme IIC; CoA, coenzyme A.

b The values shown are means (standard errors) (×10^−5^).

c The *P* value for W addition from split-plot ANOVA was shown. (A total of 200 SOM degradation genes were observed in this study; only genes with *P* < 0.10 were shown; for clarity, the *P* values for block, N addition, the interactive effect of block and N addition, and the interactive effect of N and W addition were not shown.)

d The abundance was increased (↑) and decreased (↓) by W addition.

10.1128/mSystems.00374-19.8 TABLE S5 Relative abundance of SOM degradation genes significantly affected by N addition (only genes with *P* < 0.10 were shown). Download Table S5, XLSX file, 0.01 MB. Copyright © 2019 Zhang et al. 2019 Zhang et al. This content is distributed under the terms of the Creative Commons Attribution 4.0 International license.

In contrast to functional gene diversity, there was no significant difference between the observed community similarity and the expected community similarity (from the null model) for the taxonomic OTU diversity for three out of four experimental treatments (*P* > 0.05; Table S4). This indicates that the stochastic processes (rather than deterministic processes) are the primary driver of microbial taxonomic diversity. Consistently, there were nonsignificant differences in the taxonomic SES values among these treatments (*P* > 0.05), indicating that the primary driver of stochastic processes was not affected by the experimental treatments.

### N addition decreased average genome size and increased AOB abundance 11-fold.

Consistent with the significant effect of N addition on the relative abundances of core community genes (Fig. 1b and d), N addition significantly decreased the average genome size of soil microbial communities (*P* < 0.05; Fig. 2b). In particular, the average genome size changed from 5.93 ± 0.03 (×10^6^ bases) under the non-N-addition treatments to 5.81 ± 0.04 under the N-addition treatments.

**FIG 3:**
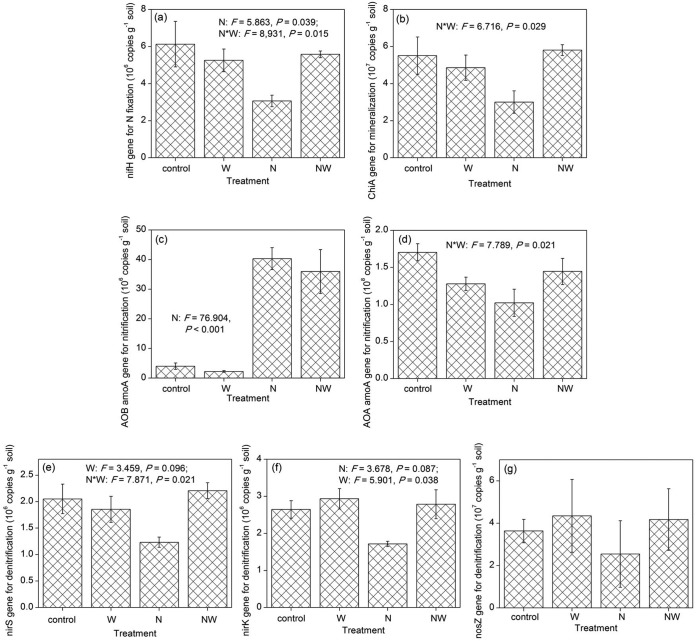
Effects of experimental treatments on the abundances of N-cycling genes of soil microbial communities. Error bars represent one standard error (four replicates). For clarity, only the statistical results with *P* < 0.10 are shown in the figure.

The N-addition treatments increased the AOB-*amoA* (for ammonia oxidization) gene abundance (from quantitative PCR [qPCR]) by ∼11.2-fold relative to the non-N-addition treatments (Fig. 3c). As a complementary piece of evidence, the AOB-*amoA* gene abundance from real-time PCR correlated significantly with both the gene relative abundances from shotgun metagenome and ammonia oxidization potential (Fig. S2). In addition, N addition decreased the absolute abundances (based on real-time PCR) of the *nifH* (N fixation), *chiA* (chitin mineralization), AOA-*amoA* (archaeal ammonia oxidization), *nirS* and *nirK* (denitrification), and *nosZ* (denitrification) genes by ∼30 to 50% (Fig. 3). In contrast, W addition had relatively small effects on these gene abundances.

10.1128/mSystems.00374-19.2 FIG S2 Pearson correlation analysis between qPCR results and metagenome results (a and b) and between potential ammonia oxidization rate and gene abundance (c). Download FIG S2, DOCX file, 0.2 MB. Copyright © 2019 Zhang et al. 2019 Zhang et al. This content is distributed under the terms of the Creative Commons Attribution 4.0 International license.

### Linkages among global change factors, ecosystem processes, and gene components.

**FIG 4:**
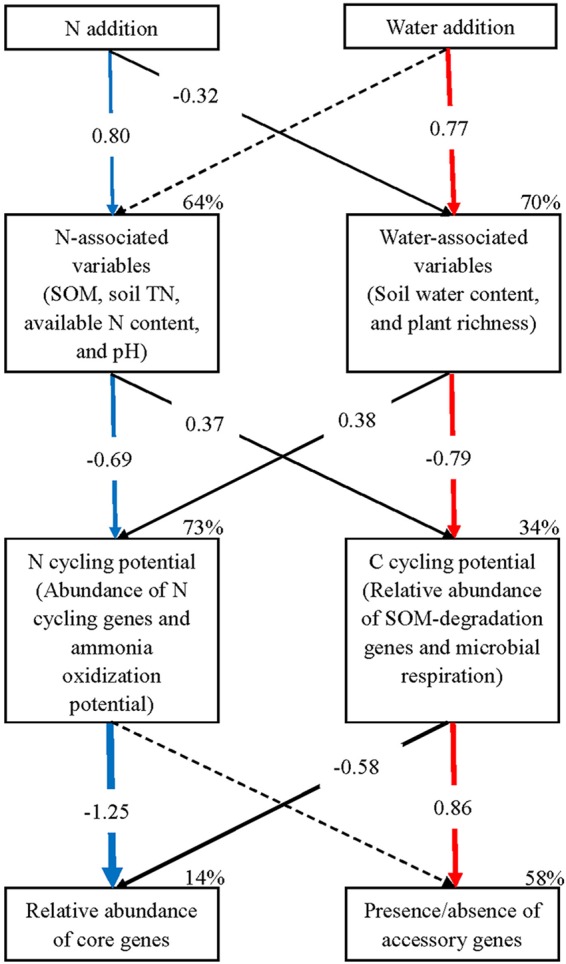
Structural equation model analysis of the effects of N and water addition on both the core and accessory genes of soil microbial communities. The final model fit the data well: χ^2^ = 10.750, *P* = 0.706, *df* = 14. Numbers in the solid arrows (*P* < 0.05) are standardized path coefficients (equivalent to correlation coefficients), while the arrow width indicates the strength of the relationships. The dashed arrows indicate nonsignificant relationships (*P* > 0.05). Percentages close to variables indicate the variance explained by the model (*R*^2^). TN, total N.

The final structural equation modeling (SEM) model adequately fit the data describing the effects of N and W addition on soil or plant variables, C- and N-cycling potentials, and both the core and accessory genes of soil microbial communities (χ^2^ = 10.750, *P* = 0.706; standardized path coefficients are given in Fig. 4). The model explained 64%, 70%, 73%, 34%, 14%, and 58% of the variation in N-associated variables, W-associated variables, N-cycling potential, C-cycling potential, the relative abundances of core community genes, and the presence or absence of accessory community genes, respectively. There were two critical linkages with large absolutes of the standardized path coefficients: one was from N addition to N-associated variables to N-cycling potential and to the relative abundances of core community genes (see blue arrows in Fig. 4), while the other was from W addition to W-associated variables (soil moisture, plant community richness) to C-cycling potential and to the presence or absence of accessory community genes (see red arrows in Fig. 4).

Consistent with the SEM results, the Mantel test indicated that microbial functional variation based on the relative abundance of core community genes was significantly correlated with both soil total N content (*r *> 0.40, *P* < 0.05) and ammonia oxidization potential (*r *> 0.22, *P* < 0.05) (Table S6). Moreover, microbial functional variation based on the presence or absence of accessory community genes was significantly correlated with microbial respiration and soil pH (*P* < 0.05; Table S6). In addition, microbial taxonomic compositional variation correlated significantly with the four indices of soil total N content, water content, soil pH, and ammonia oxidization potential (*P* < 0.05; Table S6).

10.1128/mSystems.00374-19.9 TABLE S6 Relationships between soil, plant, and microbial community indices revealed by Mantel test. Download Table S6, XLSX file, 0.01 MB. Copyright © 2019 Zhang et al. 2019 Zhang et al. This content is distributed under the terms of the Creative Commons Attribution 4.0 International license.

## DISCUSSION

### N addition affected the relative abundance of core community genes by stimulating AOB.

Both N and W were previously identified as critical limiting factors of biological diversity and ecosystem functioning in the investigated steppe ecosystem (23, 26, 27). In this study, the combination of all obtained results (Fig. 1 and 4 and Table 1) showed that experimental N addition primarily affected the relative abundances of core community genes through changing N cycling processes, while W addition primarily affected the presence or absence of accessory community genes by altering C-cycling processes, supporting our hypotheses. Furthermore, concurrent N and W addition affected both gene components through both processes (Fig. 1 and 4). A subsequent evaluation demonstrated that sequencing depth limitations did not affect these main conclusions (see details in Text S1 in the supplemental material).

10.1128/mSystems.00374-19.3 TEXT S1 Measurement of the abundance of 16S rRNA gene and seven N-cycling genes, pyrosequencing of 16S rRNA genes, calculation of the relative contribution of deterministic versus stochastic processes, statistical analysis, and the influence of sequencing depth on the results. Download Text S1, DOCX file, 0.03 MB. Copyright © 2019 Zhang et al. 2019 Zhang et al. This content is distributed under the terms of the Creative Commons Attribution 4.0 International license.

In this N-limited ecosystem, N addition fueled the activities of AOB, and thus increased the abundance of AOB-*amoA* gene 11-fold and the ammonia oxidization potential 6-fold (Fig. 3c and Table 1; see Fig. S2 in the supplemental material). All genes in the AOB genome should have shown a substantial increase in abundance similar to that of the *amoA* gene. An order of magnitude of increase in gene abundance was an extreme change compared to the relatively small changes in other genes (e.g., the other N-cycling genes; Fig. 3). Because AOB have relatively small genomes, mainly consisting of core community genes with very few accessory community genes (15), the explosive growth in AOB community members caused a decrease in the average genome size of soil microbial communities (Fig. 2b). Thus, N addition primarily affected the relative abundances of core community genes by stimulating ammonia oxidization potential (Fig. 4), which was further supported by the significant correlation between microbial functional composition based on the relative abundances of core community genes and ammonia oxidization potential (Table S6).

Although N addition directly increased soil available N content, it significantly decreased soil total N content (Table 1), which was not an expected result. The significant correlation between microbial functional variation based on gene relative abundance and soil total N content (Table S6) suggested that the changes in soil microbial communities were relatable to the decrease in soil total N content. The explosive increase in AOB population and ammonia oxidization potential should have accelerated the transformation of NH_3_ to NO_2_^−^ (and to NO_3_^−^, which is then excreted extracellularly as waste), which was easily lost from soil through leaching and denitrification (28), thus resulting in a decrease in soil total N content. NH_4_^+^-N addition could have caused a priming effect on soil total N content, which is similar to the priming effect of the addition of labile organic matter on the SOM content (29). Although the N priming effect requires further evidence and study, about 38% of previous studies observed a negative effect of N addition on soil N content (7), implying the universality of this priming effect in particular ecosystems; this topic merits further investigation.

N-addition treatments also significantly increased the SOM content (Table 1), which might be due to three reasons. First, it was very dry in the sampling year 2010 (and also in the years 2007 and 2009); therefore, N addition had little effect on plant biomass, but the combination of N and W addition caused large biomass (Table 1). Thus, N addition should have stimulated plant productivity in the past wet years (e.g., 2006 and 2008), adding more organic material into soil that remained until the year soil collection was performed in this study (26). Second, N addition decreased soil pH and thus decreased soil microbial respiration (Table 1). Third, the explosive growth of the chemoautotrophic AOB should have resulted in greater CO_2_ fixation (Fig. 3c).

Overall, most terrestrial ecosystems depend on ammonia oxidization microorganisms, which drive the major, rate-limiting step of the entire N cycle, so AOB-associated genes are very critical for the functioning of the whole ecosystem (13, 15). N addition resulted in excess N for the ecosystem, and certain forms of N such as NH_3_ are even deleterious to many organisms. Thus, AOB accelerated the transformation and transfer of N out of the ecosystem (as discussed above), helping to maintain ecosystem health.

### W addition affected the presence or absence of accessory community genes by shifting the C cycling process.

Although W addition stimulated both microbial respiration and plant richness, it had little effect on plant biomass or productivity (Table 1), which did not match our expectations and could be due to the severe arid climate conditions in this ecosystem from the years 2009 and 2010. Thus, the increase in microbial demand for C resources was not counteracted by enhanced labile C inputs from plants, leading to a limitation of the soil C resource. Consistent with this, the relative abundances of microbial genes responsible for the metabolism of plant-derived labile carbohydrates decreased under the W-addition treatments (Table 2). Furthermore, the null model method indicated that W addition significantly promoted the deterministic effect of ecological filtering in driving microbial functional gene diversity (Fig. 2a; Table S4). Consistent with the intensified ecological filtering process, W addition significantly stimulated the relative abundances of genes responsible for the degradation of amino acids and aromatic compounds (Table 2), which are generally considered more recalcitrant than plant-derived carbohydrates, indicating that W addition should primarily select for microorganisms with the pronounced ability of enhanced energy usage and diverse substrate metabolisms under instances where W does not promote plant productivity concurrently. Consistent with the evidence of gene relative abundance (Table 2), it was previously found that W addition promoted soil microbial utilization of recalcitrant SOM such as phenolic compounds, measured by Biolog EcoPlates, in another study that was conducted in the same steppe ecosystem (30). W addition favored microorganisms with genes for recalcitrant SOM degradation and accordingly selected against many other microorganisms with genes only for labile C resources. The genes for recalcitrant SOM degradation should comprise a larger proportion of the microbial genome than those for labile C resources (31). Since microbial community genome size was relatively stable (21), the microorganisms with genes primarily for the metabolism of labile C resources likely possessed a greater diversity of accessory functional genes than those with genes for recalcitrant C resources (31, 32). Thus, water addition led to a decline of total gene richness of the entire belowground microbial communities (Fig. 1a). Furthermore, the critical role of the C cycling process in determining gene richness was confirmed by the significant correlation between the microbial functional variation based on the presence or absence of accessory community genes and microbial respiration (Table S6), and it was also supported by the strong linkage between the C cycling processes and the presence or absence of accessory community genes revealed by the SEM analysis (Fig. 4). In summary, W addition primarily affected the presence or absence of accessory community genes by altering C cycling processes.

W addition should have decreased soil oxygen content and thus stimulated the abundance of the *nirK* gene involved in denitrification (Fig. 3f), which could have contributed to the marginally significant increase in soil pH (Table 1), an association that has been confirmed previously (33). Moreover, microbial functional variation based on the presence or absence of accessory community genes significantly correlated with soil pH (Table S6), suggesting that the elevated soil pH also played a role in determining gene richness. In fact, soil pH was identified to be the most important ecological factor structuring soil bacterial communities in previous studies (34, 35). In addition to the direct, primary mechanism of moisture content governing C cycling variables/potential, the elevated soil pH should be a secondary mechanism by which W addition affected the presence or absence of accessory community genes.

Because functional gene richness is primarily determined by the presence/absence of accessory community genes rather than the relative abundances of core genes, research efforts should pay more attention to the accessory community genes in order to maintain the functional diversity of soil microbial communities. Furthermore, here the C-associated variables (e.g., the amount of carbon resources) were found to be the most important factors determining the functional gene diversity, consistent with previous studies (12). Thus, with the background of multifactorial global changes, more attention should be paid to C-cycling-associated factors such as the decrease in aboveground vegetation coverage, and strategies which could elevate plant productivity and the amount of carbon resources could be adopted to effectively maintain functional gene diversity. The increase in the amount of carbon resources would relieve the pressure of the deterministic processes of ecological filtering on microbial community assembly, and accordingly stimulate the contribution of the stochastic processes of microbial migration/colonization, and thus promote microbial functional gene diversity (9, 36).

In addition, it should be noted that our conclusion indicated only that N addition had a larger effect on core community genes than accessory community genes and that W addition had a larger effect on accessory community genes than core community genes. If the magnitude of N or W addition was sufficiently large, both the core and accessory community genes may respond significantly, but the relative sensitivities of core versus accessory community genes should remain consistent. For example, although the primary effect of N addition was to affect the relative abundances of core community genes (Fig. 1b and d), gene richness seemed to be higher with the treatment of N addition alone compared to the control (although nonsignificant; Fig. 1a). N addition increased the content of soil C resources (Table 1), which might further drive the increase in functional gene richness observed here. If the amount of N added increased, the increase in gene richness may eventually become significant. Consistently, although the primary effect of N addition was on core community genes and that of W addition was on accessory community genes, N and W addition still had significant interactions on the abundances of several genes, taxonomic groups, and COG categories (*P* < 0.05; Fig. 3; Tables S2 and S3). The significant interactions were likely due to the fact that N and W addition had opposite effects on soil pH (Table 1). As the magnitude of N and/or W addition increases, more significant interactions on microbial indices would likely be observed.

### Difference between taxonomic and functional gene diversity.

Although the taxonomic composition and functional composition of soil microbial communities were closely correlated (as shown by the Mantel test), there were still three differences between the responses in the taxonomic OTUs and the functional gene indices. First, gene richness was more sensitive than OTU richness (Fig. 1 and Fig. S1), suggesting that differences in taxonomic structure do not fully correspond to differences in the functional structure, which is likely because there were many microbial species within the same OTU with >97% 16S rRNA sequence similarity in possession of unique functional traits. This has been shown previously in studies investigating cooccurring population with >97% 16S rRNA gene similarity in soils (37). Second, among all soil or plant variables, the taxonomic compositional variation most closely correlated with soil water content (*r *= 0.390, *P* = 0.003; Table S6). In contrast, none of the functional gene indices were significantly correlated with soil water content. This could be because microbial species share their genetic material through the unique route of horizontal gene transfer (38); therefore, taxonomic composition and functional composition were structured by different soil factors. Finally, the observed similarity and the expected similarity (from the null model) for the functional structure showed at least a marginally significant difference under all four treatments (*P* < 0.10; Table S4); however, the similarities for the taxonomic structure showed no difference under most treatments, suggesting that the deterministic process of ecological filtering primarily affected the functional structure rather than the taxonomic structure.

### Conclusions.

This study successfully identified critical linkages among global change factors (N deposition or fertilization and increased precipitation), ecological processes (C and N cycling processes), and community functional gene components (core and accessory community genes). Similar to the observations made in this Eurasian steppe ecosystem, both C and N were key limitation factors to soil microbial communities and AOB were responsible for the rate-limiting step of N cycling processes in many other terrestrial ecosystems (39, 40). Thus, these linkages are likely universal across a variety of ecosystems, which requires further examination. Meanwhile, metagenomes contain both the nonrelic and relic DNA (41), so technologies to enrich for nonrelic DNA or for assessment of activity, such as metatranscriptomics, should be adopted to test the linkages in future studies. Furthermore, if a given global change factor has a primary effect on one of the dichotomic functional components (core and accessory community genes) across many ecosystems, this implies that it leads to consistent shifts in the overall composition or structure of soil microbial communities (42). In addition, many previous studies tried to link C-associated global change factors or ecological processes to microbial compositional variation based on OTU or gene abundances but did not observe significant linkages (43, 44). Since C-associated factors or processes were primarily linked to the presence or absence of accessory community genes, perhaps increasing attention should be given to the gene (or also OTU) richness rather than their relative abundances in future studies (45). In contrast, for the N-associated factors or processes, more attention should be focused on OTU or gene abundance rather than their richness (46).

## MATERIALS AND METHODS

### Study site and experimental design.

This study was conducted at the Duolun Restoration Ecology Station (42°02′N, 116°17′E) of the Chinese Academy of Sciences, Inner Mongolia Autonomous Region of China. The experimental site represented a typical temperate zone with a semiarid continental monsoon climate. The mean annual temperature was 2.1°C with the monthly mean temperature ranging from –17.5°C in January to 18.9°C in July. The mean annual precipitation was ∼385.5 mm, with 80% precipitation occurring from May to August. The soil was chestnut soil (Chinese classification), corresponding to the Calcis-orthic Aridisol in the U.S. Soil Taxonomy classification, with sand, silt, and clay making up 62.7%, 20.3%, and 17.0%, respectively. The mean soil bulk density was ∼1.31 g/cm^3^. This steppe ecosystem was primarily dominated by perennials, including *Agropyron cristatum*, *Allium bidentatum*, *Artemisia frigida*, *Cleistogenes squarrosa*, *Potentilla acaulis*, and *Stipa krylovii*.

The field experiment lasted from 2005 to 2010. The effects of N, W, and their combinations were investigated. There were four blocks. Within each block, two 44 m × 28 m plots were established, with a 1-m buffer zone between the two plots. N addition or control was randomly assigned to one of the two plots. N was added in the form of urea in 2005 and NH_4_NO_3_ in 2006 to 2010 at a rate of 10 g N m^−2 ^year^−1^, conducted on a rainy day in the middle of July every year. This N addition level is within the range of airborne N deposition observed in northern China (47). N added at such a rate could persist for several months or years in this semiarid ecosystem, and thus had an accumulative effect (48). Two 10 m × 15 m subplots were set up in both the control and N amendment plots, with one plot not watered and the other plot being watered in the summer (July and August). Within each watered plot, six sprinklers were evenly arranged into two rows with 5 m between rows and 5 m between adjacent sprinklers. Each sprinkler covered a circular area with a diameter of 3 m, and thus, the six sprinklers covered the 10 m × 15 m subplot. Water (15 mm) was applied every week; thus, an additional ∼120 mm water was added each year, which corresponds to 30% of the annual mean precipitation at the study site. Overall, there are four replicates for each of the four treatments: control, N addition, W addition, concurrent N and W addition (NW).

### Measurement of plant, soil physicochemical, and microbial indices.

In late August of 2010 (the time with the highest plant biomass), aboveground vegetation was sampled by clipping all plants at the soil surface using a 0.3-m^2^ quadrant randomly placed in the subplot. All living vascular plants were sorted into species, and were oven dried at 65°C for 48 h, and weighed. The number of species detected was used to represent plant richness, and the dry mass of all living plants was used to represent the aboveground plant biomass and community composition. Meanwhile, four soil cores (10 cm deep, 3.5-cm diameter) were collected from each subplot at random and thoroughly homogenized to help circumvent small-scale heterogeneity often witnessed in soil communities (49, 50). After roots and stones were removed using a 2-mm sieve, part of the soil sample was frozen at –20°C for DNA extraction, part was kept fresh at 4°C for the measurement of inorganic labile N (NH_4_^+^-N and NO_3_-N) content, water content, microbial respiration, and ammonia oxidization potential, and the remaining portion was kept dry for the measurement of SOM content, total N content, and soil pH. SOM and total N content were quantified by the potassium dichromate-vitriol oxidization method and the Kjeldahl acid-digestion method, respectively (51). Soil NH_4_^+^-N and NO_3_-N contents were determined on a FIAstar 5000 analyzer (Foss Tecator, Denmark) after extraction of fresh soil with 1 mol/liter KCl (51). Soil water content was determined as the weight loss after drying for 24 h at 105°C. Soil pH was measured in 1:2.5 (wt/vol) suspensions of soil in distilled water.

Microbial respiration was measured by the alkali absorption method (52). Briefly, the fresh soil (20 g dry weight equivalent) was incubated in a 500-ml glass flask at 25°C under dark conditions. The glass flask was connected to a glass tube (6 cm in diameter), in which 5 ml of 50 mM NaOH solution was injected to capture CO_2_ evolved from the soil. After 4 days of incubation, the respired CO_2_ was determined by titrating the residual OH^−^ with a standardized HCl solution.

Ammonia oxidization potential was quantified by the chlorate inhibition soil slurry method as previously described with small modifications (53), which represent soil ammonia oxidation activity incubated with adequate substrate within 24 h. Adjustment of the pH of the soil slurry to 7.1 was thought to limit the activity of acidophilic ammonia-oxidizing strains (54); here in this study, ammonia oxidization potential was measured at natural pH without pH adjustment. In brief, three subsamples (5 g of fresh soil) of each sample were incubated in 50 ml falcon tubes containing 20 ml of 1 mM (NH_4_)_2_SO_4_. Potassium chlorate was added to the tubes at a final concentration of 10 mg ml^−1^ to inhibit nitrite oxidation. The suspension was incubated at 25°C for 24 h in the dark, and nitrite was extracted with 5 ml of 2 M KCl and determined with a spectrophotometer at a wavelength of 540 nm with *N*-(1-naphthyl) ethylenediamine dihydrochloride (55). The increase in the concentration of nitrite in the 24 h was calculated to represent the ammonia oxidization potential. The result was only a rough estimation of ammonia oxidization potential, because this method did not take into consideration the potential reduction process from nitrate to nitrite, although it was often negligible.

Soil DNA was extracted with the MoBio PowerLyzer PowerSoil DNA isolation kit according to the manufacturer’s instructions. To obtain sufficient DNA and to overcome the experimental constraints of soil habitat heterogeneity (50), four or five extraction replicates were conducted for each sample (0.25 g soil per extraction), and the resulting DNA extracts were pooled. The abundance of bacterial 16S rRNA gene and seven key N-cycling genes in the soil (*nifH*, *chiA*, AOB-*amoA*, AOA-*amoA*, *nirS*, *nirK*, and *nosZ*) were quantified by the method of real-time PCR (see Text S1 in the supplemental material).

### Metagenomic sequencing and microbial taxonomic and functional analyses.

In order to prepare DNA libraries for sequencing, DNA extracts were processed according to the description of the Illumina Paired-End Prep kit protocol. DNA was sheared mechanically, size selected to ∼180 bp, and gel purified. Sequencing was performed on an Illumina Hiseq 2000 platform located at the Shanghai Majorbio Bio-pharm Technology Co., Ltd. Shotgun sequencing resulted in 11.4 ± 1.3 million sequences (mean ± 1 standard error), or 2.3 ± 0.4 giga base pairs (Gbp) of sequencing effort, per sample (average of 9.2 Gbp per treatment; 36.4 Gbp total employed in this study) (Table S7).

10.1128/mSystems.00374-19.10 TABLE S7 Information pertaining to sequences merge ability and number of annotations made for shotgun metagenome data sets representing each soil sample. Download Table S7, XLSX file, 0.01 MB. Copyright © 2019 Zhang et al. 2019 Zhang et al. This content is distributed under the terms of the Creative Commons Attribution 4.0 International license.

To improve the reliability and quality of subsequent analysis, the raw sequence data were processed with the following two steps. First, the Seqprep software (https://github.com/jstjohn/SeqPrep) was used to remove the adapter sequences. Second, the library sickle (https://github.com/najoshi/sickle) was used to trim the reads from the 5′ end to the 3′ end using a sliding window (size 50 bp, step by 1 bp). If the mean quality of bases inside a window drops below 20, the remainder of the read below the quality threshold will be trimmed. We also discarded quality-trimmer reads that were shorter than 50 bp or containing N (ambiguous bases). Taxonomic profiling of clean reads is proceeded by BLASTn (blast+ version 2.2.31; cutoff E value, 1e−5) analysis against the small subunit (SSU) rRNA database of silva (release 119 [http://www.arb-silva.de]) (56). Taxon abundances were quantified at the levels of kingdom and phylum, according to the results of taxonomic assignment.

Bacteria were dominant among the three groups, and we further calculated its richness. 16S rRNA gene encoding metagenomic reads were searched against the 16S rDNA gene full-length sequences in the Greengenes database (May 2013 release), which were subsequently clustered into OTUs (operational taxonomic units) at the 97% threshold, using UCLUST (57) closed-reference OTU picking in QIIME (58). To exclude the influence of unequal sampling, the relatively rarer OTUs with <1/892 relative abundance (there were 892 16S rDNA fragments assigned to OTUs in the smallest sample) in each sample were removed for the calculation of OTU richness. To confirm the result from these metagenomic reads, we further adopted 454 pyrosequencing to quantify the OTU richness (Text S1).

Paired reads of shotgun metagenomic sequences were merged with FLASH using default parameters (Table S7) (59). Using MBLASTX, merged reads were aligned against the protein sequences downloaded from the STRING database (E-value cutoff, 1e−6; https://stringdb-static.org/download/protein.sequences.v11.0.fa.gz) (60, 61). The count matrices of COG function terms were made to summarize the results of alignment using the association file between the protein entries and COG terms (62), which was then normalized by the size of the data set (number of total annotations). To exclude the influence of unequal sampling, the relatively rarer COG genes with <5.0 × 10^−7^ relative abundance in each sample (there were >2.0 × 10^6^ reads assigned to COG genes for each sample) were removed for the calculation of COG richness (12), which was used to represent the functional richness of soil microbial communities. These COGs have been clustered into dozens of categories (there are a total of 25 COG categories); for each COG category, we also calculated its relative abundance and functional gene richness.

Merged reads were also aligned against the protein sequence of the KEGG database using MBLASTX (Table S7) (E value cutoff, 1e−6) (63), and the count matrices of KEGG orthologies (KOs) (KEGG terms) was also calculated. To estimate the SOM degradation and N-cycling potential of soil microbial communities, we focused on the genes responsible for the degradation of plant sugar materials (e.g., cellulose, chitin, poly-, oligo-, di-, and monosaccharides), amino acids, and aromatic compounds, and N-cycling genes. A total of 200 SOM degradation genes and 31 N-cycling genes were identified in this study.

The average genome size of soil microbial communities of each sample was estimated using MicrobeCensus 1.1.0 (64) after removing the adapter sequences. The parameters was set as the default (options, -n 2000000, -l 100, -q -5).

A null model method was adopted to identify the relative contribution of deterministic and stochastic processes in driving soil microbial assembly (see details in Text S1). Various statistical methods, including split-plot analysis of variance (ANOVA), permutational multivariate ANOVA (PERMANOVA) and structural equation modeling (SEM), were adopted to analyze the effects of experimental treatments on microbial functional gene diversity and composition, soil and plant variables, and their relationships (see details in Text S1).

### Data availability.

The Illumina shotgun metagenome data sets have been deposited in MGRAST with the project name of “Multifactorial Environmental Changes in Inner Mongolia of China.”
