# Supplementary material for: Distinct Drivers of Core and Accessory Components of Soil Microbial Community Functional Diversity under Environmental Changes
Source: mSystems. 2019 Oct 1;4(5):e00374-19. doi: 10.1128/mSystems.00374-19 (PMC6774018; doi:10.1128/mSystems.00374-19)
Supplement: FIG S1 [file mSystems.00374-19-sf001.docx]

**
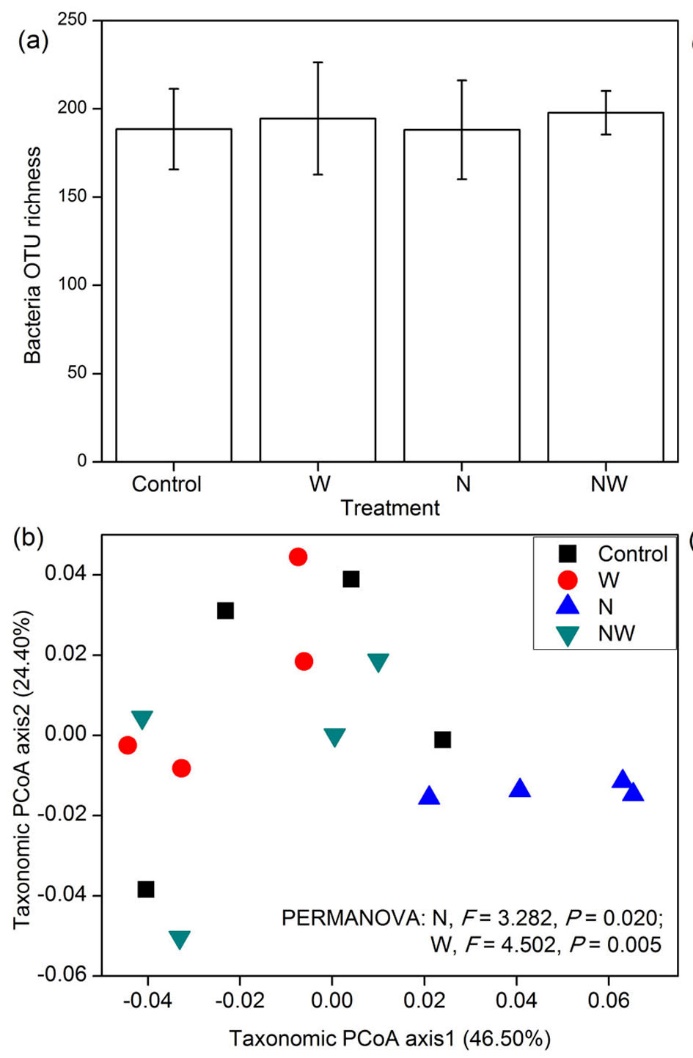

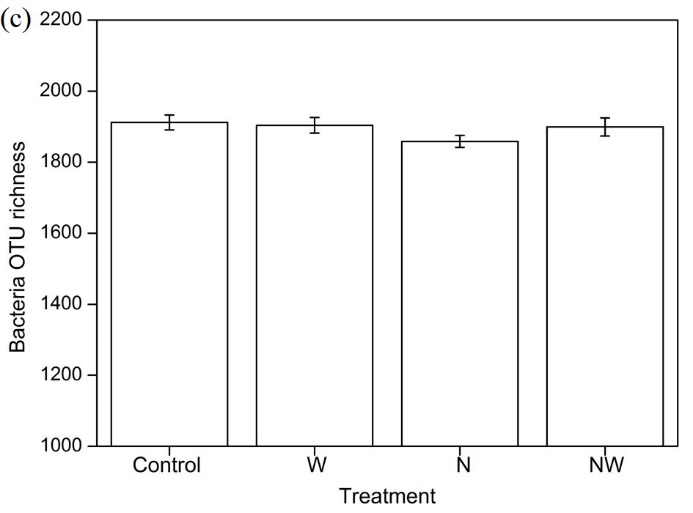
**

**Figure S1**. Effect of experimental treatments on the microbial OTU richness and composition (a, b) quantified with shotgun metagenome sequencing and the bacterial OTU richness (c) quantified with 454 pyrosequencing. Error bars represent one standard error (four replicates). The value in the bracket of the axes of Figure S1b represents the percentage of community compositional variation explained by the PCoA axes. For clarity, only the significant (*P* < 0.05) statistical results are shown in the figure.
