# Supplementary material for: Distinct Drivers of Core and Accessory Components of Soil Microbial Community Functional Diversity under Environmental Changes
Source: mSystems. 2019 Oct 1;4(5):e00374-19. doi: 10.1128/mSystems.00374-19 (PMC6774018; doi:10.1128/mSystems.00374-19)
Supplement: FIG S2 [file mSystems.00374-19-sf002.docx]

**
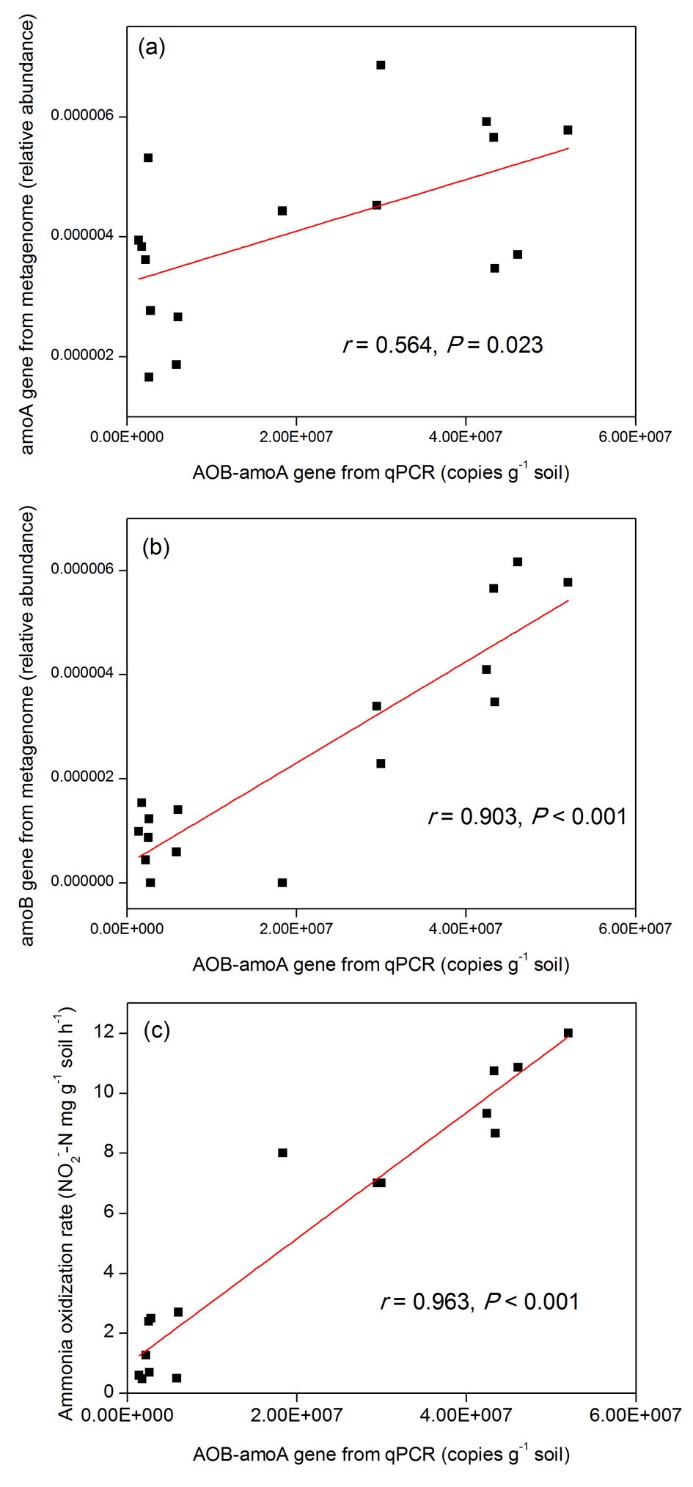
**

**Figure S2**. Pearson correlation analysis between qPCR results and metagenome results (a, b) and between potential ammonia oxidization rate and gene abundance (c).
