# Supplementary material for: Distinct Drivers of Core and Accessory Components of Soil Microbial Community Functional Diversity under Environmental Changes
Source: mSystems. 2019 Oct 1;4(5):e00374-19. doi: 10.1128/mSystems.00374-19 (PMC6774018; doi:10.1128/mSystems.00374-19)
Supplement: TEXT S1 [file mSystems.00374-19-s0001.docx]

**Method S1**

**Measurement of the abundance of 16S *rRNA* gene and seven N-cycling genes.** The abundances of 16S rRNA gene and seven N-cycling genes were quantified with real time PCR. For each gene, a standard curve was generated using a 10-fold serial dilution of a plasmid containing a copy of the target gene. The primers are: Eub338 and Eub518 for 16S rRNA gene (Zhang and Han, 2012); *nifH*-F and *nifH*-R for *nifH* gene (Rösch et al. 2002); *chif*2 and *chif* for *chif*A gene (Xiao et al. 2005); *amoA*-1F and *amoA*-2R for AOB-*amoA* gene (Rotthauwe et al. 1997); Arch-*amoAF* and Arch-*amoAR* for AOA*-amoA* gene (Francis et al. 2005); cd3AF and R3cd for *nirS* gene (Michotey et al., 2000; Throback et al., 2004); *nirK*-1F and *nirK*-5R for *nirK* gene (Braker et al. 1998); nosZ-F and nosZ-R for nosZ gene (Throback et al., 2004). The 25-µL PCR reaction mixtures contained 12.5 µL SybrGreen qPCR Master Mix (2×) (Shanghai Ruian BioTechnologies Co., Ltd., Shanghai, China), 0.5 µL each of 10 µM forward and reverse primers, 2.5 µL BSA (10 mg/mL), and 8.0 µL sterile, DNA-free water. 1.0 µL standard plasmid or soil DNA extract (1.2-5.1 ng) was added per PCR reaction. The reaction was conducted using an ABI7500 FAST Real-time PCR system. The following program was used for the 16S *rRNA* gene: 95°C for 1 min followed by 40 cycles of 95°C for 5 s, 55°C for 15 s and 72°C for 15 s. The following program was used for the genes of *nifH*, *chiA*, AOB-*amoA*, AOA-*amoA* and *nirK*: 95°C for 2 min followed by 40 cycles of 95°C for 10 s, 55°C for 20 s and 72°C for 1 min. The following program was used for the genes of *nirS* and *nosZ*: 95°C for 2 min followed by 40 cycles of 95°C for 10 s and 60°C for 1 min. Melting curve and gel electrophoresis analyses were conducted to confirm that the amplified products were the appropriate size. The equation Eff = [10^(-1/slope)^ - 1] was used to calculated the amplification efficiencies and resulted in the following values: *rRNA* 96%, *nifH* 92%, *chiA* 95%, AOB-*amoA* 87%, AOA-*amoA* 95%, *nirS* 92%, *nirK* 86% and *nosZ* 93%. The gene copy number was calculated using a regression equation that related the cycle threshold (C_t_) value to the known number of copies in the standards. For each sample, the qPCR reactions were performed in triplicate. We added BSA to the PCR reaction mixes to reduce the inhibitory effects of co-extracted polyphenolic compounds in the soil. Additionally, to estimate the possible inhibitory effects of co-extracted polyphenolic compounds, three replicates of PCR for several samples were conducted after adding known amounts of standard plasmid with the soil DNA extract. Inhibitory effects were found to be negligible.

**Pyrosequencing of 16S rRNA gene.** To confirm the result of bacterial OTU richness from these metagenomic reads, we further adopted 454 pyrosequencing to quantify the OTU richness. Primers 27F (5’-AGA GTT TGA TCC TGG CTC AG-3’) and 338R (5’-TGC TGC CTC CCG TAG GAG T-3’) were used to amplify 16S rRNA gene fragment. To measure all 16 samples in a run, a unique 10-mer tag for each sample was added to the 5’-end of the primer 338R. Each 20μl PCR mixture contained 4μl FastPfu Buffer (5×; Transgen), 2μl of 2.5mM dNTPs, 0.8μl of DNA template, 0.4μl of each primer (5μM), and 0.4μl of FastPfu Polymerase (Transgen). PCR protocol was: 95℃ for 2 m (denature); 25 cycles of 95℃ for 30 s (denature), 55℃ for 30 s (anneal), 72°C for 30 s (elongate); and 72℃ for 5 m (elongate). Three replicates of PCR were performed for each sample, after which the products were combined and purified by agarose gel electrophoresis, recovered, and quantified with PicoGreen using a TBS-380 Mini-Fluorometer. Equal molar concentrations of PCR products for each sample were then pooled and sequenced in a Roche 454 Genome Sequencer FLX Titanium system at Shanghai Majorbio Bio-pharm Technology Co., Ltd. The reads were deposited in the Sequence Reads Archive database of the National Center for Biotechnology (accession no. SRA057669). These reads were analyzed following the steps as described before [61]. Briefly, to minimize the influence of unequal sampling on the following calculated indices, we randomly selected 3,478 reads for each sample. All these sequences (3,478 × 16) were clustered into OTUs with larger than 97% similarity.

**The relative contribution of deterministic vs. stochastic processes.** β-diversity, which represents the compositional variation between communities, is often used to infer the possible mechanisms of community assembly (such as the relative importance of deterministic vs. stochastic processes). However, the difference in the β-diversity indices may be caused by differences in the ecological processes as well as α- and γ-diversity. To exclude the influence of the other two diversity components, Chase (2010) developed a null model method, which compares the observed β-diversity to the theoretical β-diversity from stochastically assembled communities. To identify the relative contribution of deterministic and stochastic processes in driving soil microbial assembly, we analyzed the community data of each treatment, following the steps of Chase (2010) and Zhou et al. (2014). First, for any given pair of plots within the treatment, we calculated the observed OTU/gene richness (e.g., α_1_ and α_2_ for plot 1 and 2, respectively) and the number of shared OTU/genes (SS_obs_). Second, the total number of OTU/genes detected in the “OTU/gene pool” (γ-diversity) from all plots of the treatment, and the proportion of the plots occupied by each OTU/gene was measured. Third, we calculated the distribution of the expected shared OTU/genes from the null model (SS_exp_) by randomly drawing α_1_ and α_2_ OTU/genes from the OTU/gene pool with the probability of an OTU/gene to be drawn proportional to its among-plot occupancy. The SS_exp_ and the expected Jaccard’s similarity (*J*_exp_) are obtained for each drawing, and the average Jaccard’s similarity (_exp_) and its SD are estimated based on 10,000 drawings (σ_exp_). For each treatment, permutational analysis of multivariate dispersions (PERMDISP) was adopted to test the difference between the observed community similarity (*J*_obs_) and the average of the expected community similarity (_exp_) (Anderson 2004). The non-significant difference (*P* > 0.05) meant that stochastic processes were the primary driver of microbial diversity, while the significant larger (or smaller) *J*_obs_ relative to _exp_ suggested that the deterministic process of ecological filtering (or competitive conclusion) was the primary driver.

To investigate whether the treatments affected the relative contribution of deterministic and stochastic processes in driving microbial diversity, the community data of all plots of all treatments was analyzed together to calculate *J*_obs_ and _exp_ (and its σ_exp_), following the same steps as stated above. In other words, here the OTUs/genes of all treatments were taken as the OTU/gene pool. The magnitude of deterministic processes on community structure was further quantified with the index of SES (standard effect size) (Kraft et al. 2011, Zhou et al. 2014): SES = (*J*_obs_ –_exp_)/σ_exp_. PERMDISP was adopted to test whether the SES values were different among the four treatments (Anderson 2004).

**Statistical analysis.** Split-plot ANOVA was used to assess the effect of experimental treatments on gene richness, abundance of N-cycling genes, and many other soil, plant, and microbial indices. The *P*-values were corrected via Bonferroni correction (Benjamini and Hochberg 1995). Bray-Curtis distances, based on the relative abundance of COG genes (or microbial phyla), were calculated to represent the functional (or taxonomic) compositional variation among samples (Bray and Curtis 1957). To identify the potential difference between core and accessory genes, Bray-Curtis distances based on the genes observed in all samples (which should primarily be the core genes) or the genes observed in only part of these samples (which should primarily be the accessory genes) were also calculated. Principal coordinate analysis (PCoA) was used to visualize relative differences among samples (Anderson 2003). Permutational multivariate ANOVA (PERMANOVA) was used to test the effect of experimental treatments on the compositional variation (Anderson 2005). The Mantel test was adopted to assess the relationships between microbial community structure and soil or plant indices or microbial process rates (Bonnet and Peer 2002). Pearson correlation was used to construct the relationship between gene abundance from qPCR and gene relative abundance from metagenome or microbial process rate. All data were tested for normal distribution prior to statistical analyses, which were performed with SPSS software (SPSS 13.0 for Windows). To analyze the effect of sequencing depth on our results, the equal sampling standard was also changed from deleting genes with < 5 × 10^-7^ relative abundance (see the aforementioned method) to deleting genes with < 1 × 10^-6^ relative abundance (to mimic a small sequencing depth).

We further adopted structural equation modeling (SEM) to gain a mechanistic understanding of how N and W addition affected the core and accessory genes of soil microbial community. SEM is based on a simultaneous solution procedure, where the residual effects of predictors are estimated (partial regressions) once common causes from inter-correlations have been statistically controlled for (Grace 2006). N addition significantly affected the contents of SOM, total N, available N and soil pH (Table 1), so the first PCoA axis of the four variables was taken as the N-associated variable in the SEM analysis. W addition significantly affected soil water content and plant richness (Table 1), so the first PCoA axis of the two variables was taken as the W-associated variable. Similarly, in the SEM analysis, the first PCoA axis of ammonia oxidization potential and the abundances of N-cycling genes (representing N-cycling rates) was taken as the N cycling potential, and the first PCoA axis of microbial respiration and the relative abundances of SOM-degradation genes (representing C-cycling rates) was taken as the C cycling potential. And the first PCoA axis from Bray-Curtis distance based on the relative abundance of core genes and that based on the presence/absence of accessory genes were also used in the SEM analysis. We started SEM analysis with the specification of a conceptual model of hypothetical relationships, based on a priori and theoretical knowledge (Figure 4). Briefly, we assumed that N and W addition will alter different soil and plant variables, which will turn to affect C and N cycling potentials and further the core and accessory genes of soil microbial communities. In the SEM analysis, we compared the model-implied variance–covariance matrix against the observed variance–covariance matrix, and the data were fitted to the models using the maximum likelihood estimation method. Adequacy of the models was determined using χ2 tests, and adequate model fits are indicated by a nonsignificant χ2 test (*P* > 0.05) (Grace 2006, Wei et al. 2013). SEM analyses were performed using AMOS 18.0 (Amos Development, Spring House, Pennsylvania, USA).

**Influence of sequencing depth on the results.** To analyze the effect of sequencing depth on our results, we have deleted genes with < 1 × 10^-6^ relative abundance to mimic a smaller sequencing depth and found that the results were similar (data not shown). In other words, sequencing depth limitations did not seem to affect the conclusion that N and W addition primarily affected the core and accessory genes of soil microbial communities, respectively. W addition selected for certain microbial taxa with increased capacity to utilize recalcitrant SOM over many others, decreasing their relative abundances. It was the decrease in their relative abundances that led to the decrease in observed gene richness. Since the decrease in the relative abundance of these taxa was independent of the sequencing depth, the decrease in gene richness should also be independent. Similarly, the observed effect of N addition on the relative abundance of core genes should also be independent of the sequencing depth.

**References**

Anderson, M. J. 2003. *PCO: a FORTRAN computer program for principal coordinate analysis.* New Zealand: Department of Statistics, University of Auckland.

Anderson, M. J. 2004. *Permutation dispersion: a FORTRAN computer program for permutational analysis of multivariate dispersions (for any two-factor ANOVA design) using permutation tests*. New Zealand: Department of Statistics, University of Auckland.

Anderson, M. J. 2005. *PERMANOVA: a FORTRAN computer program for permutational multivariate analysis of variance.* New Zealand: Department of Statistics, University of Auckland.

Benjamini, Y., and Y. Hochberg. 1995. Controlling the false discovery rate: a practical and powerful approach to multiple testing. *Journal of the Royal Statistical Society: Series B (Statistical Methodology)* 57:280–300.

Bonnet, E., and Y. V. Peer. 2002. zt: a software tool for simple and partial Mantel tests. *Journal of Statistical Software* 7:1–12.

Braker G, Fesefeldt A, Witzel KP. 1998. Development of PCR primer systems for amplification of nitrite reductase genes (nirK and nirS) to detect denitrifying bacteria in environmental samples. *Applied and Environmenatl Microbiolgy* 64: 3769–3775.

Bray, J. R., and J. T. Curtis. 1957. An ordination of the upland forest communities of southern Wisconsin. *Ecological Monograph* 27:325–349.

Chase, J. M. 2010. [Stochastic community assembly causes higher biodiversity in more productive environments](http://apps.webofknowledge.com/full_record.do?product=UA&search_mode=GeneralSearch&qid=5&SID=V1jYoOp42CaGS1QXF1H&page=1&doc=4). *Science* 328:1388–1391.

Francis CA, Roberts KJ, Beman JM, Santoro AE, Oakley BB, et al. 2005. Ubiquity and diversity of ammonia-oxidizing archaea in water columns and sediments of the ocean. *Proceedings of the National Academy of Sciences USA* 102: 14683–14688.

Grace, J. B. 2006. *Structural Equation Modeling and Natural Systems*. Cambridge, UK, New York: Cambridge University Press.

Kraft, N. J. B., L. S. Comita, J. M. Chase, [N. J](https://www.ncbi.nlm.nih.gov/pubmed/?term=Sanders%20NJ%5BAuthor%5D&cauthor=true&cauthor_uid=21940897). Sanders, [N. G](https://www.ncbi.nlm.nih.gov/pubmed/?term=Swenson%20NG%5BAuthor%5D&cauthor=true&cauthor_uid=21940897). Swenson, [T. O](https://www.ncbi.nlm.nih.gov/pubmed/?term=Crist%20TO%5BAuthor%5D&cauthor=true&cauthor_uid=21940897). Crist, … and J. A. [Myers.](https://www.ncbi.nlm.nih.gov/pubmed/?term=Myers%20JA%5BAuthor%5D&cauthor=true&cauthor_uid=21940897) 2011. Disentangling the drivers of β diversity along latitudinal and elevational gradients. *Science* 333:1755–1758.

Michotey V, Me´jean V, Bonin P. 2000. Comparison of methods for quantification of cytochrome cd1-denitrifying bacteria in environmental marine samples. *Applied and Environmental Microbiology* 66: 1564–1571.

Rösch C, Mergel A, Bothe H. 2002. Biodiversity of denitrifying and nitrogenfixing bacteria in an acid forest soil. *Applied and Environmental Microbiology* 68: 3818–3829.

Rotthauwe JH, Witzel KP, Liesack W. 1997. The ammonia monooxygenase structural gene amoA as a functional marker: molecular fine-scale analysis of natural ammonia-oxidizing populations. *Applied and Environmental Microbiology* 63: 4704–4712.

Throback IN, Enwall K, Jarvis A, Hallin S. 2004. Reassessing PCR primers targeting nirS, nirK and nosZ genes for community surveys of denitrifying bacteria with DGGE. *FEMS Microbial Ecology* 49: 401–417.

Wei, C., Q. Yu, E. Bai, [X](https://www.ncbi.nlm.nih.gov/pubmed/?term=L%C3%BC%20X%5BAuthor%5D&cauthor=true&cauthor_uid=23925948). Lü, [Q](https://www.ncbi.nlm.nih.gov/pubmed/?term=Li%20Q%5BAuthor%5D&cauthor=true&cauthor_uid=23925948). Li, [J](https://www.ncbi.nlm.nih.gov/pubmed/?term=Xia%20J%5BAuthor%5D&cauthor=true&cauthor_uid=23925948). Xia, … and X. [Han.](https://www.ncbi.nlm.nih.gov/pubmed/?term=Han%20X%5BAuthor%5D&cauthor=true&cauthor_uid=23925948) 2013. Nitrogen deposition weakens plant-microbe interactions in grassland ecosystems. *Global Change Biology* 19:3688–3697.

Xiao X, Yin X, Lin J, Sun L, You Z, et al. 2005. Chitinase genes in lake sediments of Ardley Island, Antarctica. *Applied and Environmental Microbiology* 71: 7904–7909.

Zhang X, Han X. 2012. Nitrogen deposition alters soil chemical properties and bacterial communities in the Inner Mongolia grassland. *Journal of Environmental Sciences* 24: 1483–1491.

[Zhou](http://www.pnas.org/search?author1=Jizhong+Zhou&sortspec=date&submit=Submit), J., [Y. Deng](http://www.pnas.org/search?author1=Ye+Deng&sortspec=date&submit=Submit), P. [Zhang](http://www.pnas.org/search?author1=Ping+Zhang&sortspec=date&submit=Submit), K. [Xue](http://www.pnas.org/search?author1=Kai+Xue&sortspec=date&submit=Submit), Y. [Liang](http://www.pnas.org/search?author1=Yuting+Liang&sortspec=date&submit=Submit), J. D. [Van Nostrand](http://www.pnas.org/search?author1=Joy+D.+Van+Nostrand&sortspec=date&submit=Submit), … and A. P. [Arkin.](https://www.ncbi.nlm.nih.gov/pubmed/?term=Arkin%20AP%5BAuthor%5D&cauthor=true&cauthor_uid=24550501) 2014. Stochasticity, succession, and environmental perturbations in a fluidic ecosystem. *Proceedings of the National Academy of Sciences of the United States of America* 111:E836–E845.
